# Food Availability in Different Food Environments Surrounding Schools in a Vulnerable Urban Area of Santiago, Chile: Exploring Socioeconomic Determinants

**DOI:** 10.3390/foods11070901

**Published:** 2022-03-22

**Authors:** Anna Christina Pinheiro, Daiana Quintiliano-Scarpelli, Jacqueline Araneda Flores, Claudio Álvarez, Mónica Suárez-Reyes, José Luis Palacios, Tito Pizarro Quevedo, Maria Rita Marques de Oliveira

**Affiliations:** 1 Carrera de Nutrición y Dietética, Facultad de Medicina-Clinica Alemana, Universidad del Desarrollo, Santiago 7610658, Chile; 2 PhD Program in Nursing, Medical School, São Paulo State University, Botucatu 18618-687, Brazil; mrmolive@ibb.unesp.br; 3 Faculty of Health and Food Sciences, University of Bío-Bío, Chillán 3810189, Chile; jaraneda@ubiobio.cl; 4 IT Innovation Center for Social Apps, University of Santiago de Chile, Santiago 9170020, Chile; claudio.alvarezs@usach.cl; 5 Escuela de Ciencias de la Actividad Física, el Deporte y la Salud, University of Santiago de Chile, Santiago 9170020, Chile; monica.suarez@usach.cl; 6 Center for Studies in Food Science and Technology, University of Santiago de Chile, Santiago 9170020, Chile; jose.palacios@usach.cl; 7 Faculty of Medical Sciences, University of Santiago de Chile, Santiago 9170020, Chile; tito.pizarro@usach.cl; 8 Institute of Biosciences, São Paulo State University, Botucatu 18618-689, Brazil

**Keywords:** school food environments, healthy foods, unhealthy foods, multidimensional poverty, polices

## Abstract

The analysis of the food environment is used to identify areas with gaps in the availability of healthy foods and can be used as a public policy assessment tool. In recent decades, Chile has implemented several strategies and regulations to improve food environments, with encouraging results. Little is known about the scope of these measures in socially vulnerable environments. This study is part of a project that seeks to build an integrated intervention model for healthy school environments in a vulnerable area of Santiago, Chile. The objective of this study was to evaluate the availability of healthy and unhealthy foods around schools and the relationship between it and socioeconomic determinants of the school community in the Chilean context. A cross-sectional study to measure the food environment of informal markets (street food), formal markets (stores), and institutions (schools) was conducted in and around 12 schools (100 m surrounding schools) in a vulnerable urban area of Santiago, Chile. A lack of healthy foods was observed, which was related to some socio-economic determinants and the multidimensional poverty was the most relevant. The diagnosis of food environments around schools can represent an important target for governments to implement policies focused at improving the availability of healthy foods.

## 1. Introduction

Food environments (FE) are described as the conditions that influence or enable people to access healthy and/or unhealthy foods [1,2]. Many studies identify the major FE components, such as spatial availability of foods in a defined territory or structural policies that allow the selection of certain types of foods and purchase decisions [3]. The importance of analyzing the FE is based on the possible associations between the multiple components and risk factors for obesity, chronic diseases [4,5], and food insecurity [6,7,8]. In this sense, it is important to apply instruments that can measure the particularities of the FE, considering the local context (e.g., socio-economic, culture, and food regulations) [3,9]. Previous studies have shown inequalities in access to food [1,2]. Lower-income communities tend to be surrounded by a greater number of convenience stores that sell unhealthy foods at the expense of healthier foods. In addition, a higher density of food stores that sell unhealthy food around schools compared to healthy food establishments has been reported, which may promote excess weight in students.

The approach to food and nutrition interventions or evaluations that consider an ecological model are a main target to include in a FE characterization and the food audit model is generally used to characterize the FE. Aspects such as the availability of healthy or unhealthy foods, the variety of healthy foods and prices, or the presence of advertisements are considered [10,11]. Furthermore, different studies evaluate the spatial distributions of sale points (e.g., supermarkets, corner shops, local markets) in the analysis and characterization of the FE [12,13]. This kind of graphic representation allows for the identification of areas with less availability of healthy foods and the characterization of food deserts or food swamps [13]. Thus, it is possible to develop public policies that encourage the reduction in existing gaps, primarily by considering that areas with less availability of healthy foods are the same areas as those with high levels of poverty, a high prevalence of obesity and food insecurity, and are located in more vulnerable areas [14].

In recent decades, Chile has implemented several initiatives aimed at controlling the large increases in obesity and chronic diseases. Inclusion of front of package warning labels (FOP) on foods with a high content of critical nutrients (calories, sugar, sodium, and saturated fats), which helps with the identification of unhealthy packaged foods [15], or the increased tax on sugary drinks [16] are some examples of the recent policies implemented. These policies have demonstrated positive results, such as a decrease in the content of critical nutrients on packaged foods [17,18] and, to a lesser degree, consumption of sugary drinks [19,20]. However, there is a lack of studies on the spatial behavior of these policies in the FE of different socioeconomic and cultural contexts.

Considering the serious problem of childhood obesity in many countries, it is essential to understand its causal factors for the development of public policies that can address the problem efficiently and effectively. Therefore, analyzing and modifying the FE of a school, for example, is considered one of the most effective strategies for evidence-based interventions for childhood obesity, assuming that healthy options should be the easiest and most accessible [21]. According to the Food and Agriculture Organization (FAO), “a healthy school food environment allows and encourages the school community (children, family members, school officials, etc.) to make food choices that are consistent with the healthiest diets and the installation of better well-being” [22].

In order to implement structural measures that make it possible to identify areas with a low supply of healthy foods, it is necessary to measure the FE for subsequent intervention. In developing the National Food and Nutrition Policy (PNAN), the Chilean Ministry of Health identified the most relevant FE for evaluation and intervention in the country: domestic/home, stores, restaurants, street food, and institution [23]. Considering the Chilean context, we applied metric tools (MT) that consider the FE identified in the PNAN and local food regulation laws, except the home FE which was not considered. In this work, we measured the availability of healthy and unhealthy foods in and around 12 schools in a vulnerable urban area of Santiago, Chile, and evaluated the relationship with social and economic determinants of the school community. Although an analysis of the FE considers other factors such as price, advertising, and variety, in this study we addressed the food availability dimension, responding to one of the objectives of a larger project, which is to promote the supply of healthy foods in the school environment. Our hypothesis was that there would be a low availability of healthy foods in the school FE and that these results would be influenced by socio-economic determinants.

## 2. Materials and Methods

### 2.1. Setting and Sample

This observational cross-sectional study is part of the research project named “Development, scaling, and validation of an integrated system of interventions in schoolchildren in nutrition, physical activity and community environment in Ciudad Sur” (FONDEF IT18I0016), which seeks to build an integrated intervention model for healthy school environments. Part of the intervention model includes promoting the availability of healthier foods in the school environment.

From a non-probabilistic sample, 12 public schools from six low-income municipalities of the Metropolitan Region, Chile (El Bosque, La Granja, San Ramón, Lo Espejo, San Joaquín, and Pedro Aguirre Cerda) were invited to participate. These municipalities belong to a vulnerable urban sector with high poverty and a high presence of immigrants. In Chile, the school vulnerability is measured by an index associated with poverty (range 0–100) and the average index of participating schools in our sample was 91.5 ± 5.4 (min: 88.0; max: 98.0), which reflects high vulnerability due to poverty [24]. All schools were selected by the local government and the assessment of the food environment was part of the baseline assessment of the main project FONDEF IT18I0016 (Table 1).

### 2.2. Instrument

The FE was measured using MT developed as part of the project “Exposure to unhealthy food environments and diet quality in obese and eutrophic schoolchildren in the Ñuble Region” (FONIS SA18I0127) (Table S1). All instrument creation and validation processes were part of the activities of this project (FONIS SA18I0127).

First, an extensive review of the literature was carried out to identify instruments used for the measurement of FE. Using the information collected, a first draft of the instrument was designed, considering the five Chilean FE (domestic/home, stores (corner/neighborhood stores), restaurants, street food, and institution) [23]. Next, these instruments were reviewed by two separate panels of experts from the fields of nutrition, public health, agricultural production, and public policy using focus group methodology, after which adjustments were made. Finally, a quantitative analysis (internal consistency, reliability, and construct validity) was carried out to determine the final instruments.

Although the instruments consider various dimensions of the FE (availability, diversity, advertising), this study was focused on availability of food (healthy and unhealthy foods) at the points of sale in the school FE. Three of five different MT were used, based on the FE defined for Chile in the National Police of Food and Nutrition: (1) institution (kiosks and cafeterias inside the school), (2) street food or informal markets (all mobile points of sale), and (3) stores or formal market (supermarkets, local markets, etc.) [23]. For institution FE, only observation of kiosks was considered, since more than 80.0% of schoolchildren are beneficiaries of the School Feeding Program (PAE); thus, most eat the same standardized lunch at school. The PAE provides a healthy meal, absent of foods with FOP.

The healthy food group included fruits, vegetables, dairy, pulses, meats and eggs, soy, fresh/frozen/ready-to-eat fish and/or seafood and/or shellfish, grains and processed products based on cereals with no added sugar or no “High in” front of packaging label, beverages/water/fruit nectar with no added sugar and without “High in” label, 100% fruit juices and without added sugar or without “High in” label. Unhealthy foods include products with more than one “High in” label: salty and sweet snacks, sausages and cured meat packages, ice cream, baked or fried sweet doughs containing refined sugars, fried or baked savory doughs with or without filling, fast food, soft drinks/juices/fruit nectar/sports or energy drinks processed with added sugar and “High in” label.

### 2.3. Data Collection

The food environment metric tools were applied inside the schools (institution FE) and in the surrounding areas. A radius of 100m was defined based on the possibility that schoolchildren buy food/snacks upon arrival to or departure from school during a typical school day (08:15 a.m.–4:30 p.m.). Response options were dichotomous (yes/no) for the presence/absence of each item. The MT was completed by trained nutritionists at the beginning or the end of the school day, using an observational procedure followed by data entry.

To determine the geographic coordinates (latitude and longitude) of each point of sale, a software application was developed. Data was entered into a geographic information system identifying the type of point of sale (street food, stores, institution) and QGIS version 2.18 software was used for map construction.

All data were collected between November 2019 and March 2020. We used the web application REDCap (www.https://www.project-redcap.org/, 16 February 2022) licensed by Universidad del Desarrollo to create all databases.

### 2.4. Variables and Statistical Analysis

Food availability was measured considering each positive answer (yes) from the MT as 1 point, and negative answer (no) as zero point and the presence of healthy versus unhealthy food (Δ = total healthy foods − total unhealthy foods) was calculated. There were more healthy food groups considered than unhealthy, as described below.

Each FE (stores, street food, institution) was analyzed independently. Descriptive statistics (mean and standard deviation) for the total score of healthy, unhealthy, and Δ availability of each FE were presented by food groups. Positive Δ scores indicate higher availability of healthy foods, and negative scores, higher availability of unhealthy foods. These scores were categorized according to interquartile considering their range of distribution in each FE. Scores were classified according by availability: (1) very low (score less than the 25th percentile), (2) low (score between the 25th and 50th percentiles); (3) average/intermediate (score between the 50th and 75th percentiles); and (4) high (greater than the 75th percentile). The proportion of each group by FE dimension and by school is presented in tables and figures.

Univariate and multiple linear regressions were performed to analyze the relationship between availability of healthy foods (dependent variable), social and economic determinants of the school community (prevalence of obesity), school vulnerability index, obesity rate, community development index (economy, education, wellness) [25], and multidimensional poverty [26]. Independent variables of the social and economic school community with *p*-values ≤ 0.20 were selected for a subsequent multiple regression model using a stepwise forward procedure. Homoscedasticity was tested using the Breusch–Pagan/Cook–Weisberg test. Statistical significance was set at *p* < 0.05. Stata 16.1 software (College Station, TX, USA) was used for analyses.

## 3. Results

**Figure 1:**
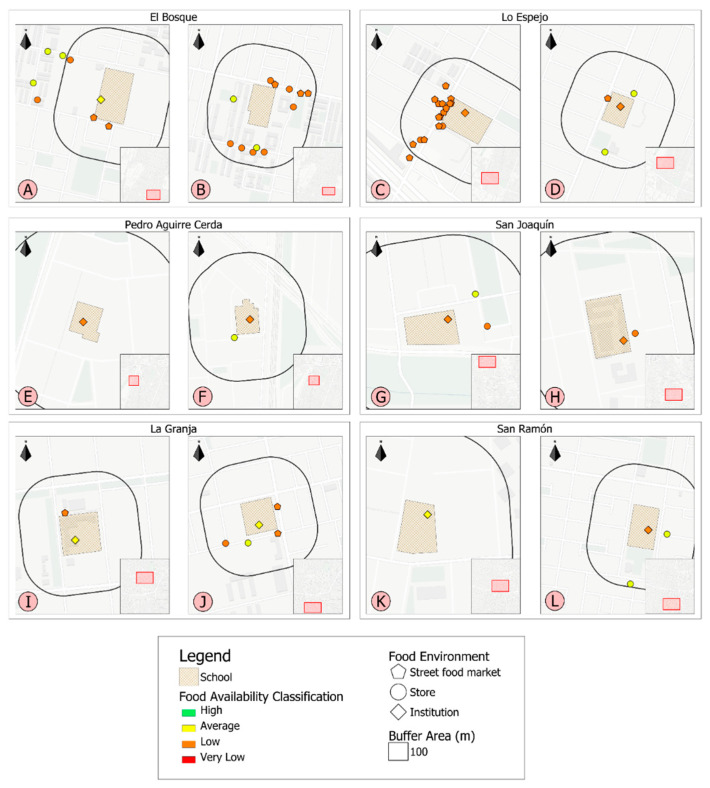
Distribution of food availability classification inside schools and the 100 m surrounding each school in the six studied municipalities.

Figure 1 shows the distribution of food availability in schools and the 100 m around each school. Every FE was classified according to food availability, from very low (red) to high (green) availability, with each school represented by a letter (A–L). In general, it low availability of healthy foods in the studied schools FE area was observed. No point of sale was classified as high availability (green) in or around schools. The municipalities “El Bosque” and “Lo Espejo” presented a higher density of food points of sale around schools (street food and stores). One school did not have an institution FE (school B) and the others presented average or low availability of healthy foods.

The possible score range for each food group and totals are presented in Table 2 (range column) and the general description of food availability scores according to the presence of each food group (healthy and unhealthy) in street food, stores, and institution environments.

Considering the availability of healthy versus unhealthy food in street food, the mean score obtained was −0.96 ± 1.9. Analyzing by food group, the highest average scores were for dairy (0.44 ± 0.8) and non-sugary beverages (1.28 ± 1.2). The availability of unhealthy foods was higher than healthy foods 4.16 ± 1.6 and 3.2 ± 2.1, respectively. The unhealthy food group with highest average score was snacks, salty and sweet, with more than one front of package warning label (FOP) (1.33 ± 1.18).

For the stores FE, the total value was 6.2 ± 3.8 for availability of healthy versus unhealthy food. The highest healthy food group values were also for dairy (3.2 ± 1.8) and non-sugary beverages (2.8 ± 1.7). For unhealthy groups they were snacks, salty and sweet, with more than one FOP (2.20 ± 0.96) and soft drinks and juices with added sugar (1.58 ± 0.76). The mean for healthy foods was 13.3 ± 5.7 and 7.1 ± 2.8 for unhealthy foods.

For the institution FE (inside schools) the mean availability of healthy versus unhealthy foods was 4.9 ± 5.6. The highest availability scores were for non-sugary beverages (2.5 ± 1.6) and prepared dishes (1.5 ± 2.2). The total healthy foods score (7.9 ± 5.6) was higher than that of unhealthy foods (3.0 ± 2.8). The most prevalent unhealthy food remained as the salty and sweet snack with more than one FOP.

A higher availability score for fruit was observed in the institution FE (1.0 ± 1.0) and for vegetables in the stores FE (0.73 ± 0.5).

Table 3, Table 4 and Table 5 describe the proportion of availability of healthy foods (very low, low, average, and high) by school according to FE (street food, stores, and institution).

The street food FE had mostly very low availability of healthy foods (96.0%) (Table 3). The availability of healthy versus unhealthy food was low in 100% of schools, showing that the presence of unhealthy foods exceeds the presence of healthy foods. None of the schools had average or high availability of healthy foods in the street food FE. Nevertheless, the analysis of the components in the instrument to measure FE indicates that the presence of total healthy foods only reached 11.93% of the maximum scale (0–29), dairy reached 7.3% (0–6), fruits reached 6.5% (0–4), and non-sugary beverages 21.3% (0–6) (data not shown in tables or figures).

The stores FE had mostly average availability of healthy and unhealthy foods, indicating low availability of healthy versus unhealthy foods (Table 4). Although availability of total healthy foods reached 47.5% of the maximum scale (0–28), dairy reached 58.3% (0–6), fruits reached 13.5% (0 to –4), vegetables reached 73.0% (0–1), and non-sugary beverages reached 56.0% (0 to –5) (data not shown in tables or figures).

For the institution FE, we observed mostly low and average classifications for availability of healthy versus unhealthy foods. Four schools had an average classification (A, I, J, K), indicating that the presence of total healthy foods is higher than the presence of unhealthy foods. Total healthy foods analyzed separately were mostly very low or low and the same results were observed for unhealthy foods (Table 5). Better availability was observed for fruits, dairy, and non-sugary beverages reaching 25.0%, 16.6%, and 41.6%, respectively, of the maximum expected range (data not shown in tables or figures).

Thus, the total availability of healthy foods (street food, stores, and institution) in the analyzed schools was 27.2% of the maximum expected range.

Table 6 shows the associations between the availability of healthy versus unhealthy foods (Δ-availability) and social and nutritional variables related to the school and municipality. In the univariate model, the variables obesity rate, community development index, and multidimensional poverty were significantly associated with availability of healthy versus unhealthy foods. In the multivariate model, only multidimensional poverty index remained significantly related. For each decrease of 0.37 points in multidimensional poverty index (range 0–1), there was an increase of one point in the Δ-availability (ß −0.37; CI 95%: −0.67; −0.08). This model explained 12% of the variance in the dependent variable.

## 4. Discussion

The school FE in the southern neighborhoods of Santiago, Chile, present unsatisfactory availability of healthy foods. This lack of availability related to socio-economic determinants, with multidimensional poverty being particularly important. These results were in accordance with our hypothesis. Previous evidence also shows that vulnerability relates to a lower supply of healthy foods in and surrounding schools, with a high presence of food deserts and food swamps [14]. It is interesting to note that even within an area considered vulnerable, there are differences, evidenced by the instrument used. This was the first study to use the instrument developed to measure the FE considering the Chilean context (local regulations, food pattern consumption, types of food stores).

The results indicate that the instrument was sensitive to environmental variations. Therefore, an increase in the supply of healthy foods, or a decrease in the supply of unhealthy foods, can change the score attributed to the point of sale and change its classification. Furthermore, it is interesting to mention that these metric tools consider a greater weight for healthy foods compared to unhealthy foods. As the availability of unhealthy foods is so high, even with this lower attributed weight, the low availability of healthy foods is evident.

Another important issue to consider is the perimeter around the schools. Studies mapping FE generally consider determine a perimeter between 100 and 500 m for evaluation purposes. Considering this aspect, identifying and classifying FE becomes important when designing, implementing, and evaluating public policies that seek to increase the consumption of healthier foods by the population. Various efforts have been made to develop and validate instruments that can provide a correct reading of FE and, subsequently, be used to test associations with health conditions and individual and community socioeconomic factors [11,14,27]. These studies have been developed with instruments adapted to the social and cultural context of each country and have shown the low availability of food in the surroundings of schools.

In some countries, household FE present stores selling low quality and a poor variety of fruits and vegetables and a high availability of ultra-processed products contributing to elevated prevalence of overweight [28]. In the school environment, Carmo et al., (2018) [29] found a greater exposure to obesogenic environments in private schools compared to public schools, both inside and around them. In another study, the presence of stores selling fruits and vegetables in the neighborhood was associated with higher fruit and vegetable consumption [30]. Similar results were found in the United States where higher neighborhood poverty and higher fruit and vegetable prices were associated with lower fruit and vegetable intake [31].

Although there are similarities in the description of FE in various countries according to the social and economic determinants of health, it is important to highlight that most studies that have characterized FE have been carried out in high-income countries. Therefore, extrapolating results to low- and/or middle-income countries may introduce bias, since the structural conditions of urban spaces and food purchases are different [3]. For example, in high-income countries, the presence of food swamps, geographic areas with a high density of corner stores, predicts obesity better than the presence of food deserts [32]. In low- and middle-income countries, neighborhood food outlets, unlike corner stores, offer fresh, unpackaged food, often derived from local farmer’s markets.

In the last decade, Chile has implemented regulations that aimed to protect children against unhealthy food environments. Some regulations have received a strong international recognition on obesity control, such as Law 20,606 [15]. This law, implemented in 2016, includes the presence of front-of-package warning labels for foods high in critical nutrients. The law also includes the prohibition of advertising focused on children and the sale of products high in critical nutrients at schools [15]. Although some studies have shown a decrease in the supply of unhealthy foods in schools [33], our results indicate that there was low availability of healthy foods. However, it is important to highlight that we studied school environments in economically and socially vulnerable neighborhoods. As neighborhoods with those characteristics are a priority for public policies, our results are even more important.

In Chile, the Ministry of Health stimulates local governments to create local regulations aimed to restrict the sale of unhealthy foods around schools. In our sample, just one municipality (schools I, J) implemented this regulation. Our mapping data shows a high density of food points of sale around some schools, mainly schools B and C. Most of them were street foods, principally food hawkers that move to school surroundings at the beginning and end of the school day.

Very low and low availability of healthy foods were observed in the street food points of sale evaluated. No store presented a high availability of healthy foods, rather an average level of healthy food availability predominated.

The results of this study emphasize the strong presence of an obesogenic environment in the school environments of the analyzed neighborhoods, which related to multi-dimensional poverty. Similar results have been observed in the Netherlands, in which it was shown that unhealthy food options (snacks, sugar-sweetened beverages) were more available for sale in comparison with healthy options (fruits, vegetables, bottled water) with few differences by socio-economic factors [34]. A national study in New Zealand also demonstrates an important obesogenic environment (density of convenience stores) near schools with access to unhealthy foods mainly in urban areas and close to the most deprived urban schools [35]. Although the presence of an obesogenic environment, with low availability of healthy foods, can relate to health outcomes such as obesity [32], instruments for measuring the FE such as ours are not capable of determining or identifying consumption. Thus, it is necessary to complement studies with other tools, such as food surveys supported by digital platforms [36] or methodologies that have a qualitative approach to the problem [3].

Obesity has increased independently of the social and economic conditions of the population, and, in this study, multidimensional poverty was associated with less healthy environments. This represents a great challenge for policies, as educational processes will not be enough to change the behavior of families, underlying the importance of investing in favorable environments.

Despite weak evidence relating school FE with health outcomes, these results highlight the presence of an obesogenic environment in and around the studied schools. It was expected that there would be few associations between the analyzed variables and the food environment, as the work was carried out in neighborhoods with similar characteristics.

Although several regulations have been implemented in Chile to reduce the exposure of schoolchildren to unhealthy foods, it is necessary to continue advancing in initiatives that promote the increase in the supply of healthier foods in the school environment. Additionally, greater oversight by health authorities is necessary to enforce current regulations. On the other hand, it is important to engage the school community, participating both in complaints of non-compliance and in the joint construction of solutions for the implementation of healthier environments for schoolchildren. It was expected that there were few associations between the analyzed variables and the food environment, given the similar characteristics between neighborhoods.

The fact that the instrument used was created specifically for our local context and that the study focused on low-income communities may limit the ability to extrapolate results. On the other hand, there is a need for instruments sufficiently adaptable for different regional contexts and for studies that deepen our understanding of particular realities, especially in neighborhoods with greater social and economic vulnerability.

This study has some limitations, for example, the instrument used to measure food environments, if used in other countries, needs adaptations, since it considers packaged foods with front of package warning labels as unhealthy; also, a global analysis of food environments in which the schoolchildren are inserted must include the children’s home, in addition to the school environment. In addition, the evaluation of the consumer interaction with the FE should consider additional constructs, not measured by these MT, that can influence food selection, such as personal preferences [3].

## 5. Conclusions

These study findings show low availability of healthy foods around schools in vulnerable areas of southern Santiago. In addition, availability is inversely associated to multidimensional poverty. To promote healthy food environments in schools, in addition to regulations that prohibit the marketing and availability of unhealthy foods to children, we must consider the eating and purchase behavior of schoolchildren.

Furthermore, school kiosks or cafeterias compete against the cheaper foods, that are often less healthy, of hawkers when students arrive or leave school. To obtain healthy school FE, the prohibition of offering and selling of unhealthy foods inside the schools is apparently not sufficient. It is necessary to eradicate the unhealthy food offered within and around schools and work towards generating a supply of attractive healthy foods at lower prices inside schools. More studies are needed to analyze how to improve environments in terms of increasing the availability of healthy foods and making foods more affordable, in order to reduce obesity. In addition, a review of the most suitable fiscal measures for school settings is necessary in order to make greater access to healthy food a reality.

It is necessary to continue searching for the best strategies in the evaluation of FE to allow for the implementation of evidence-based interventions that can positively impact the health of the population. Studies that evaluate the geographic availability of foods are a useful tool to identify gaps for public health policies.

Future studies should incorporate information about the habits and purchasing power of families into the audit and evaluation presented here, which may contribute to the identification of additional actions to ensure student health and well-being and reinforce the policies that Chile has implemented in recent decades to improve the nutrition of the population.

## Figures and Tables

**Table 1:** Sample description according to municipality, school, and food environment type.

Municipality	School ID	Food Environment Type Distribution			Total
		Street Food	Stores	Institution	
		n	n	n	n
El Bosque	A	2	5	1	8
	B	3	9	0	12
Lo Espejo	C	16	2	1	19
	D	1	2	1	4
Pedro Aguirre Cerda	E	0	0	1	1
	F	0	1	1	2
San Joaquín	G	0	2	1	3
	H	0	1	1	2
La Granja	I	1	0	1	2
	J	2	2	1	5
San Ramón	K	0	0	1	1
	L	0	2	1	3
**Total**		**25**	**26**	**11**	**62**

**Table 2:** General description of food availability scores according to presence of each food group in different food environments.

Availability	Street Food		Stores		Institution	
	Range	$\bar{X}\pm \mathrm{SD}$	Range	$\bar{X}\pm \mathrm{SD}$	Range	$\bar{X}\pm \mathrm{SD}$
**Food Groups**						
Fruit	0–4	0.26 ± 0.6	0–4	0.54 ± 1.0	0–4	1.00 ± 1.0
Vegetables	0–1	0.08 ± 0.3	0–1	0.73 ± 0.5	0–1	0.20 ± 0.4
Dairy	0–6	0.44 ± 0.8	0–6	3.20 ± 1.8	0–6	1.00 ± 1.0
Legumes	0–1	0.04 ± 0.2	0–1	0.70 ± 0.5	0–1	0.0 ± 0.0
Meat and eggs	0–4	0.16 ± 0.4	0–4	1.90 ± 1.0	0–4	0.20 ± 0.4
Grains	0–4	0.16 ± 0.5	0–4	1.00 ± 1.0	0–4	0.70 ± 0.8
Non-sugary Beverages	0–6	1.28 ± 1.2	0–5	2.80 ± 1.7	0–6	2.50 ± 1.6
Others	0–3	0.28 ± 0.5	0–3	2.30 ± 0.9	0–3	0.70 ± 0.8
Prepared Dishes	0–6	0.48 ± 0.5	-	-	0–11	1.50 ± 2.2
**Total Healthy Foods**	**0–29**	**3.20 ± 2.1**	**0–28**	**13.30 ± 5.7**	**0–29**	**7.90 ± 5.6**
Snacks with more than one FOP or sold bulk (salty and sweet)	0–3	1.33 ± 1.18	0–3	2.20 ± 0.96	0–3	1.18 ± 1.08
Sausages with more than one FOP	0–1	0.0 ± 0.0	0–1	0.69 ± 0.47	0–1	0.0 ± 0.0
Cookies and crackers with more than one FOP	0–1	0.48 ± 0.51	0–1	0.84 ± 0.37	0–1	0.45 ± 0.52
Sauces with more than one FOP (salty and sweet)	0–3	0.61 ± 0.80	0–2	0.72 ± 0.46	0–4	0.18 ± 0.40
Ice creams with more than one FOP	0–1	0.13 ± 0.34	0–1	0.63 ± 0.49	0-1	0.27 ± 0.47
Baked or fried sweet/salty dough with or without filling	0–2	0.58 ± 0.62	0–2	0.31 ± 0.54	0–2	0.29 ± 0.46
Soft drinks and juices with added sugar and energy drinks	0–2	0.87 ± 0.56	0–2	1.58 ± 0.76	0–2	0.36 ± 0.67
Fast food	0–1	0.16 ± 0.37	0–1	0.13 ± 0.34	0–1	0.27 ± 0.47
**Total Unhealthy Foods**	**0–14**	**4.16 ± 1.6**	**0–13**	**7.10 ± 2.8**	**0–15**	**3.00 ± 2.8**
**Δ Availability Healthy Foods**	**−14 to 29**	**−0.96 ± 1.9**	**−13 to 28**	**6.20 ± 3.8**	**−15 to 29**	**4.90 ± 5.6**

FOP: front of package warning label; SD: standard deviation.

**Table 3:** Classification of street food availability overall and by school.

Availability Classification	Overall	School ID					
		A	B	C	D	I	J
	%	%	%	%	%	%	%
**Healthy Foods**							
Very low	96	100	100	93.8	100	100	100
Low	4	0	0	6.2	0	0	0
Average	0	0	0	0	0	0	0
High	0	0	0	0	0	0	0
**Unhealthy Foods**							
Very low	28	0	66.7	25	0	0	50
Low	72	100	33.3	75	100	100	50
Average	0	0	0	0	0	0	0
High	0	0	0	0	0	0	0
**Δ Availability of Healthy Foods**							
Very low	0	0	0	0	0	0	0
Low	100	100	100	100	100	100	100
Average	0	0	0	0	0	0	0
High	0	0	0	0	0	0	0

Δ = Total healthy foods − Total unhealthy foods. Schools A and B: El Bosque municipality; schools C and D: Lo Espejo municipality; schools I and J from La Granja municipality.

**Table 4:** Classification of stores’ food availability overall and by school.

Availability Classification	Overall	School ID								
		A	B	C	D	F	G	H	J	L
	%	%	%	%	%	%	%	%	%	%
**Total Healthy Foods**										
Very low	19.2	40	22.2	0	0	0	0	100	0	0
Low	19.2	0	33.3	50	0	0	0	0	50	0
Average	61.6	60	44.4	50	100	100	100	0	50	100
High	0	0	0	0	0	0	0	0	0	0
**Unhealthy Foods**										
Very low	15.4	40	11.1	0	0	0	0	0	50	0
Low	15.4	20	11.1	0	50	0	0	100	50	0
Average/Intermediate	57.7	20	77.8	50	0	100	100	0	0	100
High	11.5	20	0	50	50	0	0	0	0	0
**Δ Availability Healthy Foods**										
Very low	0	0	0	0	0	0	0	0	0	0
Low	53.9	40	77.8	100	0	0	50	100	50	0
Average	46.1	60	22.2	0	100	100	50	0	50	100
High	0	0	0	0	0	0	0	0	0	0

Δ = Total healthy foods − Total unhealthy foods. Schools A and B: El Bosque municipality; schools C and D: Lo Espejo municipality; school F: Pedro Aguirre Cerda municipality; schools G and H: San Joaquín municipality; school J: La Granja municipality; school L from San Ramón municipality.

**Table 5:** Classification of institution (inside schools) food availability overall and by school.

Availability Classification	Overall	School ID										
		A	C	D	E	F	G	H	I	J	K	L
	%	%	%	%	%	%	%	%	%	%	%	%
**Total healthy foods**												
Very low	63.6	0	100	0	100	100	100	100	0	100	0	100
Low	27.3	100	0	100	0	0	0	0	100	0	0	0
Average	9.1	0	0	0	0	0	0	0	0	0	100	0
High	0	0	0	0	0	0	0	0	0	0	0	0
**Unhealthy Foods**												
Very low	63.6	0	0	0	0	100	100	100	100	100	0	100
Low	27.3	100	100	0	100	0	0	0	0	0	100	0
Average	9.1	0	0	100	0	0	0	0	0	0	0	0
High	0	0	0	0	0	0	0	0	0	0	0	0
**Δ Availability Healthy Foods**												
Very low	0	0	0	0	0	0	0	0	0	0	0	0
Low	63.6	0	100	100	100	100	100	100	0	0	0	100
Average	36.4	100	0	0	0	0	0	0	100	100	100	0
High	0	0	0	0	0	0	0	0	0	0	0	0

Δ = Total healthy foods − Total unhealthy foods. School A: El Bosque municipality; schools C and D: Lo Espejo municipality; schools E and F: Pedro Aguirre Cerda municipality; schools G and H: San Joaquín municipality; schools I and J: La Granja municipality; and schools K and L from San Ramón municipality.

**Table 6:** Association of availability of healthy versus unhealthy foods with environmental variables from the school and municipality.

Variables	Univariate			Multivariate Model		
	ß (95% CI)	*p*-Value	Adjusted R^2^	ß (95% CI)	*p*-Value	Adjusted R^2^
School Vulnerability Index	0.06 (−0.19; 0.31)	0.650	−0.01			0.12
Obesity Rate	−0.09 (0.22; 0.05)	0.190	0.01	0.01 (−0.08; 0.28)	0.274	
Community Development Index	56.9 (19.1; 94.7)	0.004	0.13			
- Economics	55.3 (3.42; 107.1)	0.037	0.06	−9.15 (−87.3; 69.03)	0.816	
- Education	31.7 (9.1; 54.3)	0.007	0.10			
- Wellbeing	81.8 (−13.8; 177.4)	0.585	0.03			
Multidimensional Poverty	−0.26 (−0.42; −0.01)	0.002	0.13	−0.37 (−0.67; −0.08)	0.013	

CI: confidence interval.

## Data Availability

Data is contained within the article (or supplementary material).

## Supplementary Materials

The following are available online at https://www.mdpi.com/article/10.3390/foods11070901/s1, Table S1: Description of the foods considered in the availability evaluation food environment metric tool, by each studied dimension: stores, street food and institution.
