# Supplementary material for: Food Availability in Different Food Environments Surrounding Schools in a Vulnerable Urban Area of Santiago, Chile: Exploring Socioeconomic Determinants
Source: Foods. 2022 Mar 22;11(7):901. doi: 10.3390/foods11070901 (PMC8997463; doi:10.3390/foods11070901)
Supplement: Supplementary file 1 [file foods-11-00901-s001.zip › foods-1623057-supplementary.pdf]

Table S1

Description of the foods considered in the availability evaluation food environment metric tool, by each studied dimension: stores, street food and institution.

**Classification of the availability/existence**	**Type of food environment**
	Stores
Healthy foods<br>*(Positive score: 1 point for each food group)*	- Fruits: dried and/or fresh fruits packed or not without added sugar and/or salt, frozen fruits.<br>- Vegetables: fresh/packed/frozen.<br>- Dairy: low fat (liquid or powder), without FOP "High in" and added sugar.<br>- Pulses: fresh beans, lentils, chickpeas and/or peas (frozen, ready-to-eat, dried).<br>- Meats and eggs: low-fat meats (fresh, frozen, ready-to-eat, low in sodium), texturized soy, fish and/or seafood (fresh, frozen, ready-to-eat, low in sodium), eggs (boiled).<br>- Cereals: breakfast cereals with no added sugar or no "High in" FOP, cereals (quinoa, amaranth, whole rice), whole wheat bread, ready-to-eat sandwich with whole wheat bread (with vegetables and / or dairy), sweet doughs and/or industrialized low or no sugar pastry products.<br>- Beverages/Water: bottled water (natural and/or flavored with no added sugar and without "High in" FOP), fruit juices and beverages (no added sugar or without "High in" FOP.<br>- Others: vegetable oil (olive, canola, sunflower, other); non-nutritive sweeteners (inulin, sucralose, stevia, others).
Unhealthy foods<br>*(Negative score: 1 point for each food group)*	Products with more than one "High in" FOP:<br>- Salty snacks (French fries, other processed foods).<br>- Sweet snacks (cookies, chocolates, cakes).<br>- Sausages and cured meats packaged.<br>- Sauces and dressings (ketchup mayonnaise, mustard, etc.).<br>- Sweet Sauces (Nutella®, ice cream sauces).<br>- Ice creams.<br>- Fast food (pizzas, hot dog, hamburger.<br>- Soft drinks/ juices with added sugar.<br>- Energy drinks.<br>Sweet snacks sold in bulk (not packaged).
Street food	
Healthy foods<br>*(Positive score: 1 point for each food group)*	- Fresh fruits.<br>- Juice fruit and nuts with no added sugar.<br>- Fresh vegetables without dressings.<br>- Packaged foods without or maximum 1 FOP "High in": dairy, soft drinks, flavored water, ice cream, cookies and/or snacks sweet or salty.<br>- Sandwiches and/or local dishes prepared instantly without addition of ingredients with FOP "High in" (industrialized hamburger, sausage, etc.).

Unhealthy foods<br>*(Negative score: 1 point for each food group)*	Products with more than one "High in" FOP:<br>- Salty/sweety snacks.<br>- Sauces, ice cream, soft drinks, juice fruits, sport/energetic drinks.<br>- Baked or fried sweet dough containing added sugars.<br>- Fried or baked salty dough with or without filling.<br>- Fast food (pizza, hot dog, french fries, sandwiches).
Institution (Cafeteria, Kiosk, Restaurant, etc.)	
Healthy foods<br>*(Positive score: 1 point for each food group)*	No added sugars, salt and/or without FOP "High in":<br>- Fresh/dried fruits.<br>- Fruit juices.<br>- Fresh vegetables without added dressing.<br>- Dairy.<br>- Soft drinks and flavored bottled water.<br>- Ice creams.<br>Without or maximum one FOP "High in":<br>- Sandwiches and/or local dishes prepared instantly.<br>- Snacks with or without filling, sweet or salty.<br>- No added sugar in fresh fruit juices.
Unhealthy foods<br>*(Negative score: 1 point for each food group)*	More than one FOP "High in":<br>- Salty or sweet snacks and cookies.<br>- Sauces and dressings.<br>- Sweet sauces.<br>- Ice creams.<br>- Soft drinks and bottled juice fruits.<br>Baked or fried sweet dough containing added sugars.<br>Fried or baked savory dough with or without filling.<br>Fast food (pizzas, hot dog, french fries, sandwiches)
